# Dataset from a mesocosm experiment on brownification in the Baltic Sea

**DOI:** 10.1016/j.dib.2022.108755

**Published:** 2022-11-17

**Authors:** Kristian Spilling, Eero Asmala, Noora Haavisto, Lumi Haraguchi, Kaisa Kraft, Anne-Mari Lehto, Aleksandra Lewandowska, Joanna Norkko, Jonna Piiparinen, Jukka Seppälä, Mari Vanharanta, Anu Vehmaa, Pasi Ylöstalo, Timo Tamminen

**Affiliations:** a Marine Research Centre, Finnish Environment Institute, Helsinki, Finland; b Centre for Coastal Research, University of Agder, Kristiansand, Norway; c Tvärminne Zoological Station, University of Helsinki, Hanko, Finland; d Geological Survey of Finland, Espoo, Finland

**Keywords:** HuminFeed, Brackish water, Runoff, Eutrophication, Plankton, Primary production

## Abstract

Climate change is projected to cause brownification of some coastal seas due to increased runoff of terrestrially derived organic matter. We carried out a mesocosm experiment over 15 days to test the effect of this on the planktonic ecosystem. The experiment was set up in 2.2 m^3^ plastic bags moored outside the Tvärminne Zoological Station at the SW coast of Finland. We used four treatments, each with three replicates: control (Contr) without any manipulation; addition of a commercially available organic carbon additive called HuminFeed (Hum; 2 mg L^−1^); addition of inorganic nutrients (Nutr; 5.7 µM NH_4_ and 0.65µM PO_4_); and a final treatment of combined Nutr and Hum (Nutr+Hum) additions. Water samples were taken daily, and measured variables included water transparency, organic and inorganic nutrient pools, chlorophyll a (Chla), primary and bacterial production and particle counts by flow cytometry.

**Specifications Table**

**Table:** 

Subject	Environmental Science
Specific subject area	Marine Ecology
Type of data	Table Figure
How the data were acquired	A mesocosm experiment at the SW coast of Finland set up in 2.2 m^3^ plastic bags treated with addition of HuminFeed (Humintech GmbH, Grevenbroich, Germany) and inorganic nutrients. Dissolved inorganic nutrients were determined by colorimetric methods using a photometric analyzer (Thermo Scientific Aquacem 250) and a spectrophotometer (Hitachi U-1100). Particulate nutrients and chlorophyll a by filtration and subsequent analysis of the filters either colorimetrically, by an element analyser (Shimadzu TOC-VCPH) or mass spectrometer (Europa Scientific ANCA-MS 20-20 15N/13C). Dissolved inorganic carbon (DIC) measurements were done using a DIC Analyzer (Model AS-C3; Apollo SciTech, USA), with a LICOR LI-7000 CO_2_ analyzer (LI-COR, USA). Colored dissolved organic matter absorption aCDOM(λ) were determined over the range of 200 to 800 nm using a spectrophotometer (Shimadzu UV-2450). Different groups of phytoplankton were determined by flowcytometry (Sysmex- Partec Cube 8), and the primary and bacterial production by incubation with radiolabeled isotopes and the uptake determined with a scintillation counter (Wallac Win Spectral 1414).
Data format	Analyzed
Description of data collection	Water samples were taken daily with a Limnos water sampler (Hydro-Bios) from 1.5 m depth from the middle of the bags. All measurements were done with this water, for example further processed by filtration or direct measurements of chemistry (e.g. concentration of inorganic nutrients) and biology (e.g. counts of plankton).
Data source location	• Country: Finland • Latitude and longitude of the experiment: 59.843N; 23.259E
Data accessibility	Repository name: Mendeley Data Data identification number: DOI: 10.17632/8ssgdbc7jt.4 Direct URL to data: https://data.mendeley.com/datasets/8ssgdbc7jt/4
Related research article	Spilling K, Asmala E, Haavisto N, Haraguchi L, Kraft K, Lehto A-M, Lewandowska A, Norkko J, Piiparinen J, Seppälä J, Vanharanta M, Vehmaa A, Ylöstalo P, Tamminen T. (2022) Brownification affects phytoplankton community composition but not primary productivity in eutrophic coastal waters: a mesocosm experiment in the Baltic Sea. Sci Tot Env 841: 156510

## Value of the Data

• Brownification is an ongoing trend in many lakes, streams and coastal regions and experimental data on the effects of this is needed to better understand what effect this has on aquatic ecosystems.
• The data will primarily benefit researchers interested in the effects of increasing dissolved organic carbon on coastal plankton communities, primary production, and heterotrophic processes.
• The data can be used for meta-analysis of brownification in different aquatic ecosystems, and these data come from a eutrophic, coastal ecosystem with brackish water.

## 1 Data Description

**Fig. 1:**
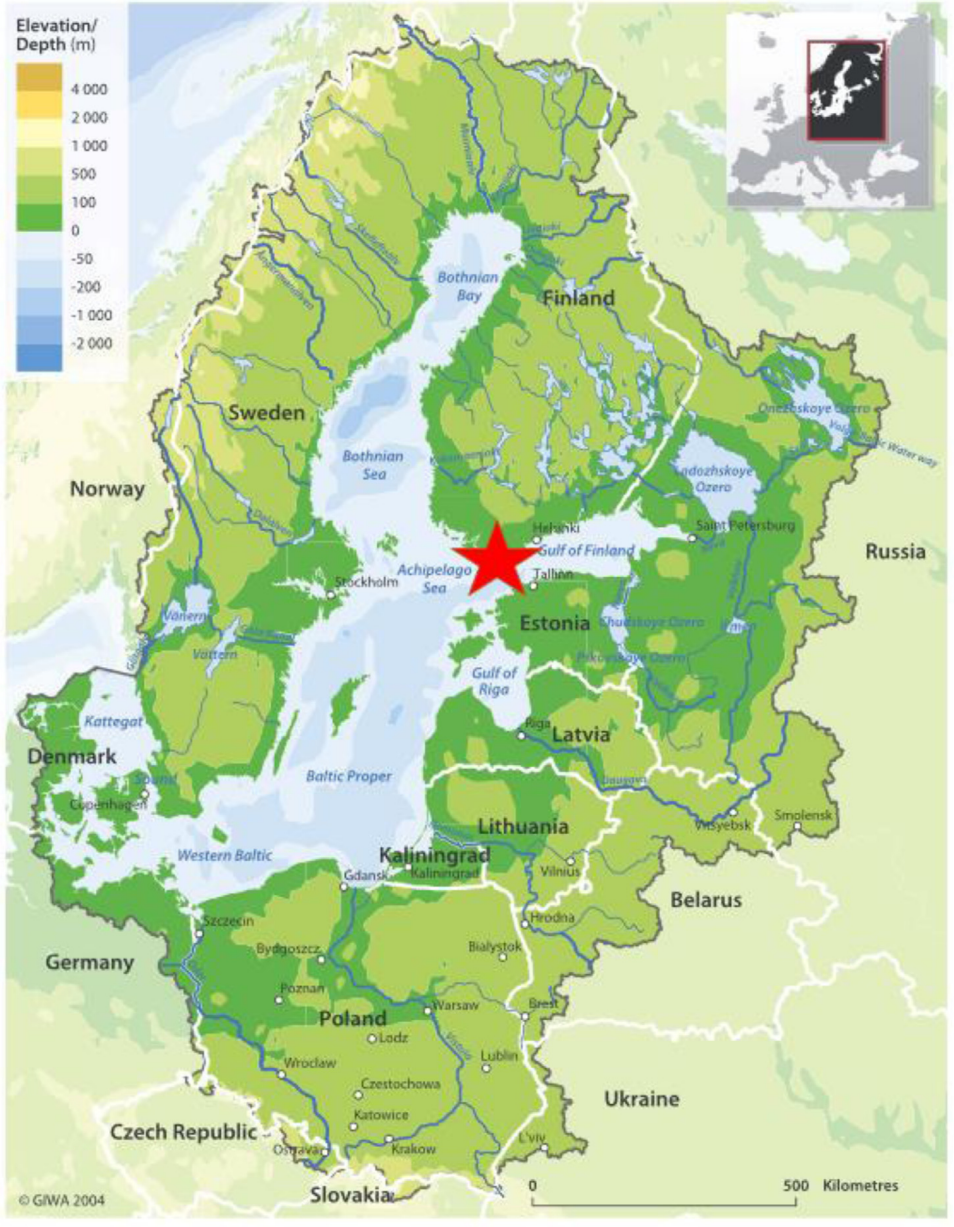
The red star marks the location of the experiment on the south-west coast of Finland. Published with permission from the copyright holder: Global International Waters Assessment (GIWA).

**Fig. 2:**
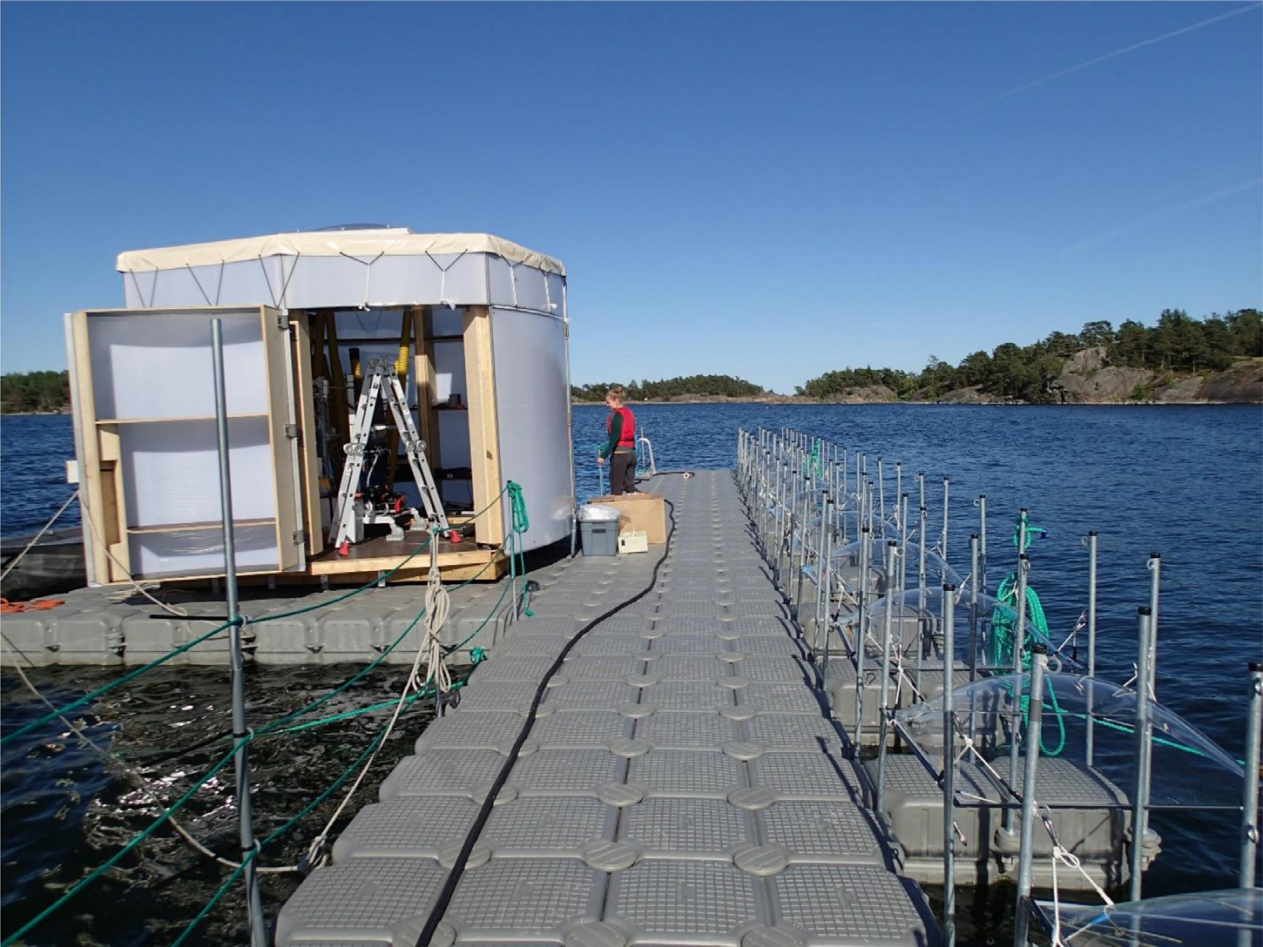
The platform before mounting the twelve mesocosms bags on the right-hand side. There were additionally two ‘dummy’ bags, one on each end to create the same light climate for mesocosm bags 1 and 12 respectively. The hut on the left-hand side houses the pumping station with real time measurements (AquaBox) that takes in samples consecutively from the mesocosms and runs the sample through an array of different sensors measuring e.g. dissolved gasses and fluorescence properties (Table 3). Photo: Kristian Spilling

**Fig. 3:**
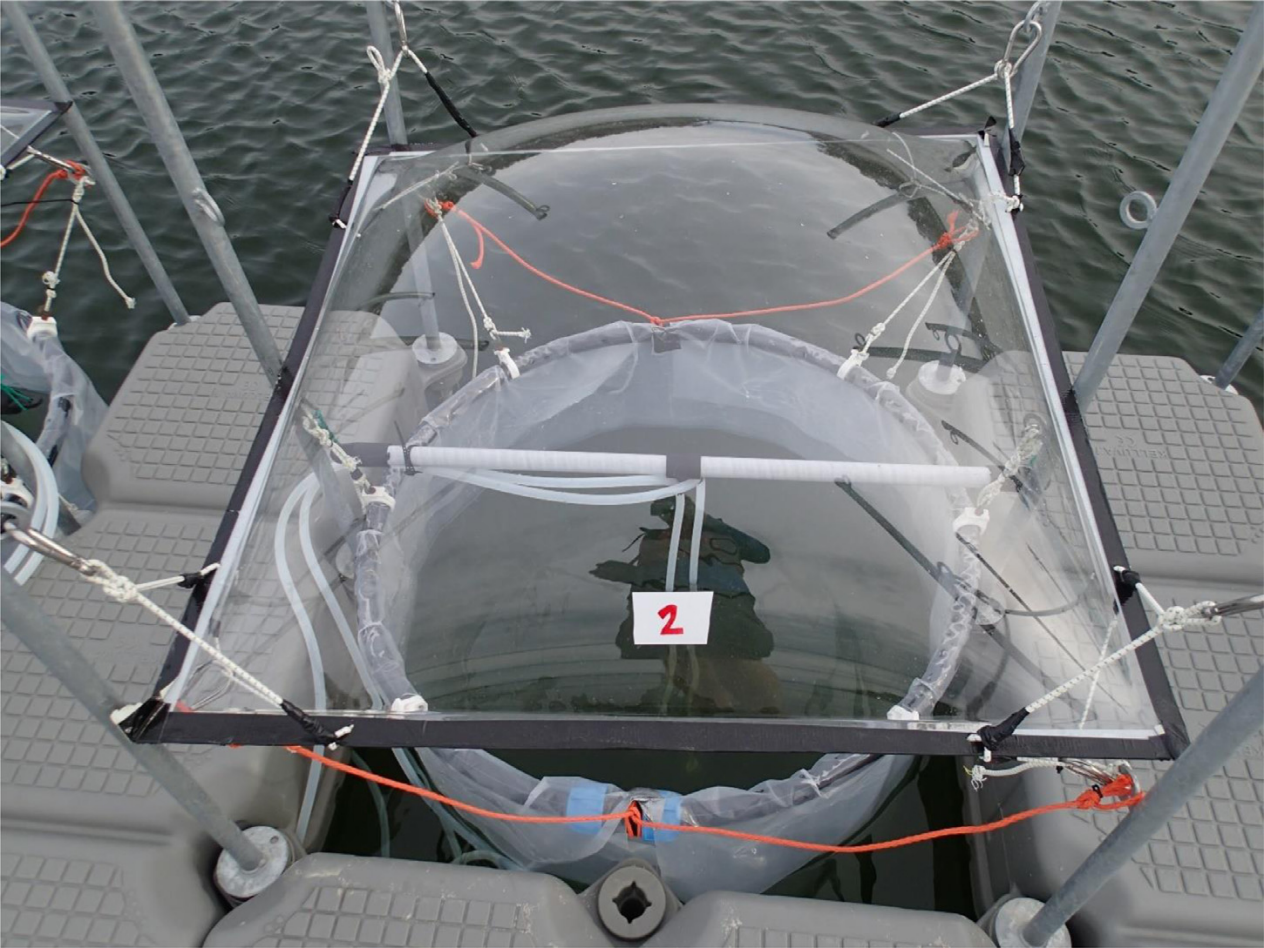
Mesocosm bag number 2 (Contr, Table 1). The tubes that go down into the middle of the bag were the sampling tubes for the automatic measurement system, AquaBox (Table 3). The lid on top prevents rainwater and debris from entering the mesocosms. Photo: Kristian Spilling

This paper contain data from a mesocosm experiment in the Baltic Sea carried out to understand how brownification and inorganic nutrients affect plankton ecology. The experiment was carried out on the SW coast of Finland (Fig. 1) close to the Tvärminne Zoological station, University of Helsinki. The mesocosm bags (2.2 m^3^) were set up with four different treatments (Table 1): control without additions (Contr), brownification with added HuminFeed (Hum), addition of inorganic nutrients (Nutr) and a combination of inorganic nutrients and HuminFeed (Nutr+Hum). Each treatment had three replicates, and the 12 bags were moored next to a floating platform (Figs. 2 and 3).

**Table 1:** The experimental treatments with the four treatments, a control (Contr) without additions, HuminFeed addition (Hum), addition of inorganic nutrients (Nutr) and a combination of inorganic nutrients and HuminFeed (Nutr+Hum).

Mesocosm	Treatment
1	Contr
2	Contr
3	Contr
4	Hum
5	Hum
6	Hum
7	Nutr
8	Nutr
9	Nutr
10	Nutr+Hum
11	Nutr+Hum
12	Nutr+Hum

All the discretely measured variables and sampling timepoints are presented in Table 2. The data are available at: https://data.mendeley.com/datasets/8ssgdbc7jt/4. This data file consists of a table with 30 columns and a description of the variables is found in Table 3. Empty cells indicate no data. The primary data is discussed in [15], and additional results on photochemical degradation of dissolved organic matter is presented in [16].

**Table 2:** The metadata of the measured variables and time points of measurements. Inorganic nutrients include NO_3_+NO_2_, NH_4_, PO_4_ and DSi, particulate nutrients POC, PON, POP and BSi, dissolved organic nutrients include DOC, DON and DOP. A full description of units are presented in Table 3 and the data can be found in the Mendeley data repository (https://data.mendeley.com/datasets/8ssgdbc7jt/4).

	Experiment day															
	0	1	2	3	4	5	6	7	8	9	10	11	12	13	14	15
Inorganic nutrients		x		x			x		x		x			x		x
Particulate nutrients		x		x			x		x		x			x		x
Dissolved organic nutrients		x		x			x		x		x			x		x
Dissolved inorganic carbon		x		x			x		x		x			x		x
CDOM absorption		x		x			x		x		x			x		x
Chlorophyll a (Chla)	x	x	x	x	x	x	x	x	x	x	x	x	x	x	x	x
Size fractionated Chla		x		x			x		x		x			x		x
Phytoplankton community	x	x	x	x	x	x	x	x	x	x	x	x	x	x	x	x
Zooplankton community		x														x
Primary production		x	x	x	x	x	x	x	x	x	x	x	x	x	x	x
Bacterial production		x	x	x	x	x	x	x	x	x	x	x	x	x	x	x

**Table 3:** The columns in the data file (https://data.mendeley.com/datasets/8ssgdbc7jt/4) with description and units. Empty cells indicate no data.

	Column	Description	Unit
1	Date	day.month.year	
2	Mesocosm	Mesocosm bag number	
3	ExpDay	Experimental day number	
4	NO3+NO2	Nitrate and nitrite concentration	µmol L^−1^
5	NH4	Ammonium concentration	µmol L^−1^
6	PO4	Phosphate concentration	µmol L^−1^
7	DSi	Dissolved silicate concentration	µmol L^−1^
8	POC	Particulate organic carbon	µmol L^−1^
9	PON	Particulate organic nitrogen	µmol L^−1^
10	POP	Particulate organic phosphorus	µmol L^−1^
11	BSi	Biogenic silicate	µmol L^−1^
12	Chla-tot	Total chlorophyll a (Chla) concentration	µg L^−1^
13	Chla_0.7-2.0	Chla fraction 0.7 – 2.0 µm	µg L^−1^
14	Chla_2-10	Chla fraction 2 – 10 µm	µg L^−1^
15	Chla>10	Chla fraction >10 µm	µg L^−1^
16	DIC	Dissolved inorganic carbon	mmol L^−1^
17	DOC	Dissolved organic carbon	mmol L^−1^
18	TDN	Total dissolved nitrogen	mmol L^−1^
19	TDP	Total dissolved phosphorus	µmol L^−1^
20	TN	Total nitrogen	µmol L^−1^
21	TP	Total phosphorus	µmol L^−1^
22	FC-Micro	Microphytoplankton	cells mL^−1^
23	FC-Nano	Nanophytoplankton	cells mL^−1^
24	FC_Syne	Synechococcus-like cells	cells mL^−1^
25	FC-Chry	Cryophyte-like cells	cells mL^−1^
26	FC-pico	Picoeukaryotes	cells mL^−1^
27	PE alpha	Initial slope of the PE curve	µmol C L^−1^ h^−1^ (µmol photons m^−2^ s^−1^)^−1^
28	PE max	Maximum photosynthesis	µmol C L^−1^ h^−1^
29	PP-model	Modelled primary production	g C m^−2^ d^−1^
30	BP-THY	Bacterial production (thymidine method)	µmol C L^−1^ h^−1^

**Fig. 4:**
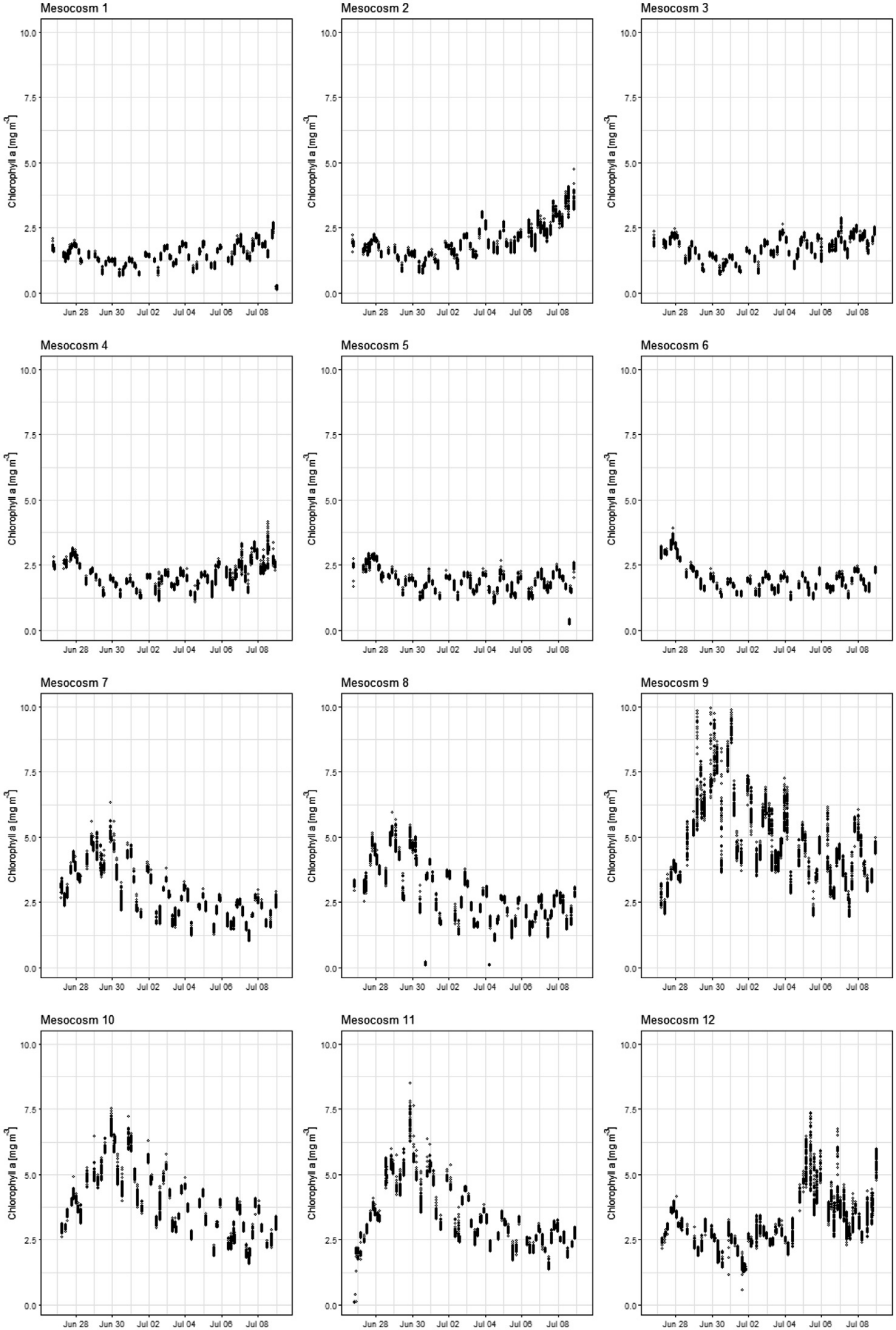
An example of the Aquabox data. Here is the raw data of Chlorophyll a (Chla) fluorescence converted to mg Chla m^−3^ by calibration against known Chla standards. The x-axis indicates the date during the experiment in 2019. The treatments in the individual mesocosms are described in Table 1.

In addition to discrete measurements, there were continuous measurements of dissolved gasses, different fluorescence properties, pH, salinity, temperature and particle images by the AquaBox (Table 4), which is a flow through measurement system developed at the Finnish Environment Institute. As an example of the data, Chla is presented in Fig. 4, and all of the data are available at https://data.mendeley.com/datasets/8ssgdbc7jt/4.

**Table 4:** The present list of instruments connected to the AquaBox. An example of the data can be found in Fig. 4 and the data from optical measurements (photosynthetic pigments, colored dissolved organic matter, and photochemical efficiency) and temperature and salinity in: https://data.mendeley.com/datasets/8ssgdbc7jt/4.

Variable	Sensor / manufacturer
Chlorophyll a fluorescence	Trios Nanoflu
Phycocyanin fluorescence	Trios Nanoflu
DOM fluorescence	Trios Nanoflu
Dissolved oxygen	Aanderaa 4330
Phycoerythrin fluorescence	Chelsea Unilux
CO_2_ partial pressure	Contros FT/Kongsberg
Thermosalinograph	Seabird SBE45
pH	Sunburst AFT
Pulse Amplitude Modulation fluorescence	PSI AquaPen
Fast Repetition Rate fluorometry (FRRF)	Chelsea FastTrack
Imaging FlowCytobot (IFCB)	McLane

## 2 Experimental Design, Materials and Methods

### 2.1 Set-up

The experiment was set up close to Tvärminne Zoological Station (59.843N; 23.259E) on the southwestern coast of Finland (Fig. 1). We used round, plastic mesocosm bags with a diameter of 90 cm and 3 m deep (Figs. 2 and 3). A total of 14 bags with a volume of 2.2 m^3^ each were attached on a row to a floating platform. Two of the bags, the ones furthest to each of the sides were filled but not used in the experiment. The purpose of these ‘dummy’ bags were to give the same light conditions for bag number 1 and 12 on each side respectively. The bags were filled 25 June 2019 (Experiment day 0, Table 2) and the treatments added the following morning, and the experiment lasted for 15 days.

Four treatments, including a control, were used, each with three replicates, totaling 12 bags (Table 1): control (Contr), brownification (Hum), inorganic nutrient addition (Nutr) and combined inorganic nutrient addition and brownification (Nutr+Hum). For the brownification treatment, a final concentration of 2 mg L^−1^ of HuminFeed, equivalent to 0.86 mg DOC L^−1^ (Meinelt et al. 2007), was added and the inorganic nutrient additions had final concentrations of 5.7 µmol NH_4_ L^−1^ (as NH_4_Cl) and 0.65 µmol PO_4_ L^−1^ (as KH_2_PO_4_).

### 2.2 Sampling

Daily water samples were taken with a Limnos water sampler (Hydro-Bios) from 1.5 m depth from the middle of the bags. A control CTD profile was taken daily from the water column outside the experimental units. A multitude of physical, chemical and optical parameters were monitored in addition to manual daily sampling with the novel flowthrough sampler Aquabox (Table 4).

### 2.3 Nutrients

We used standard colorimetric methods [1] using a photometric analyzer (Thermo Scientific Aquacem 250) to measure dissolved inorganic nutrients: nitrite + nitrate (NO_2_ + NO_3_), phosphate (PO_4_) and dissolved silicate (DSi). Ammonium (NH_4_) was measured separately using a spectrophotometer (Hitachi U-1100).

Dissolved organic carbon (DOC) and nitrogen (DON) were determined from filtered samples (0.2 µm) using a TOC analyzer (Shimadzu TOC-VCPH) equipped with a chemiluminescence detector (Shimadzu TNM-1) for total dissolved nitrogen (TDN) quantification. For determining dissolved organic phosphorus (DOP), total phosphorus (TP) was determined colorimetrically from filtered samples (0.2 µm) and DOP was calculated by subtracting the phosphate concentration from TP. Specific protocols with detailed instructions for the analyses can be found in [2], [3], [4], [5], [6].

Particulate organic carbon (POC), nitrogen (PON) and phosphorus (POP) were determined by filtration. Duplicate water samples were filtered onto acid-washed (2M HCl for 15 min then rinsed carefully with ultrapure water) and pre-combusted (450°C, 4 h) GF/F filters (Whatman). POC and PON were measured with an element analyzer coupled with a mass spectrometer (Europa Scientific ANCA-MS 20-20 15N/13C). POP was determined according to [7] modified by [5]. Samples for biogenic silicate (BSi) were filtered on polycarbonate membrane filters (0.8 µm, DHI) [4]

Water samples for dissolved inorganic carbon (DIC) measurements were collected in glass bottles with glass stoppers. Care was taken not to include any headspace and all samples were stored in a refrigerator until the measurements. During measurements, samples were kept in a water bath at 5°C, and the DIC concentration was determined using a DIC Analyzer (Model AS-C3; Apollo SciTech, USA), with a LICOR LI-7000 CO_2_ analyzer (LI-COR, USA). The DIC analyzer was warmed up for an hour before measurements and the Peltier element cooled down to 5°C before samples were inserted. Calibrations were done against known standards prepared from reagent grade Na_2_CO_3_ after heating to 285°C for two hours. These standards were measured before each measurement time point.

### 2.4 Water transparency

Samples for colored dissolved organic matter absorption aCDOM(λ) were filtered through 0.2-µm membrane filters (pre-rinsed with sample water) into acid-washed and combusted glass ampoules. After the samples reached room temperature, the absorbance spectra of the samples were recorded over the range of 200 to 800 nm using a spectrophotometer (Shimadzu UV-2450) with ultrapure water as a reference. Absorbance spectra measured with 5 cm quartz cuvette were converted to units of absorption (m^−1^).

### 2.5 Primary and bacterial production

Primary production was determined using radiolabeled ^14^C. Sub-samples were distributed in scintillation vials and placed inside a photon-irradiance (PE) incubator (constructed by B.G. Mitchell, Scripps Institute of Oceanography, University of California, USA). Briefly, the incubator is a rectangular box (65 × 8 × 15 cm) with a halogen light source at one end. On the top of the box, there are 18 incubation chambers, distributed on the longitudinal axis, and each of these incubation chambers hold one 7 mL scintillation vial. The light is channeled along the bottom part of the box and shines up through the different incubation chambers, and the photon flux density in each chamber is adjusted with apertures. We determined the light in individual wells with a spherical photosynthesis active radiation (PAR) sensor (Waltz ULM 500). The PE incubation chambers are water-cooled and were set to incubate at *in situ* temperature. For the incubations, 2 dark and 16 light bottles were used, and the irradiance ranged from 0 to 2000 µmol photons m^−2^ s^−1^.

For the ^14^C-incubations, 500 µL of 20 µCi ^14^C mL^−1^ labeled bicarbonate solution (DHI lab, Denmark) were added to 60 mL sample volume and then the water was mixed before 3 mL was distributed into the set of scintillation vials. After an incubation period of 2 h, 200 µl 1 M HCl was added and the scintillation vials were left open for 2 days, and thereafter 4 mL Hi Safe scintillation liquid was added [8]. The radioactivity was determined directly from the scintillation vials used in the incubation with a liquid scintillation counter (PerkinElmer Inc., Wallac Winspectral 1414, Wellesley, MA, USA) and calculated as carbon incorporation knowing the added activity and the DIC concentration. The PE relationship was examined by fitting the function of [9]. We used the initial slope (α) and photosynthetic maximum (Pm) in a simple model of daily production m^−2^ using average irradiance for the time of year and the measured light attenuation. This was done as we did not have continuous measurements of irradiance and light penetration into the individual mesocosms.

Bacterial production was measured as ^3^H-thymidine incorporation using cold trichloroacetic acid (TCA) and centrifugation to remove excess ^3^H-thymidine [10,11]. Three replicates and two formaldehyde-fixed adsorption blanks (final concentration 1.85%) from each sample were spiked with 20 nM [methyl-^3^H]-thymidine (Perkin Elmer), which was determined to be a saturating concentration. Sub-samples (1 mL) were incubated in eppendorf tubes for 1.5 h in the dark at *in situ* temperature. The incubation was stopped by addition of formaldehyde (final concentration 1.85%). After two-time TCA addition and removal (2x centrifugation at 20 000 g for 10 min), we added scintillation cocktail (Instagel), and the radioactivity was measured with a Wallac Win Spectral 1414 liquid scintillation counter. The activity was converted to carbon production (µg C L^−1^ h^−1^), using a cell conversion factor of 1.4 × 10^9^ cells nmol^−1^ [12] and a carbon conversion factor of 16.75 fg C cell^−1^ [13].

### 2.6 Chlorophyll and phytoplankton community

Total chlorophyll a (Chla) was determined from each mesocosm bag daily. Duplicate water samples were filtered on GF/F filters (Whatman). On days 1, 3, 6, 8, 10, 13, and 15, additional size-fractionated Chla samples were collected by pre-filtering the samples through membrane filters with pore sizes of 2 µm and 10 µm. The Chla was extracted in EtOH, stored at -20°C, and measured with a spectrofluorometer (Varian Cary Eclipse) with excitation and emission wavelengths of 430 and 670 nm, respectively.

Flow cytometer counts were done daily with a Sysmex, Partec - Cube 8 equipped with two lasers (488 and 561 nm) and two scattering (forward and side) and three fluorescence detectors (610/30; 661/16; and 670/40, corresponding to the detection of phycoerythrin, phycocyanin, and Chla, respectively). The trigger was on Chla fluorescence (670 nm). Each sampling day, we made several measurements runs with beads and blanks to ensure correct particle counts. Size fractionations (0.8, 1, 2, 5, 10, 20 µm filters) were also used to identify the approximate size of different groups. The scattering and fluorescence properties were used to gate the different phytoplankton groups using FCS Express 6 software. Five groups were identified: picoeucaryotes: size <2 µm with only Chla, Synechococcus like cells <2 µm with phycocyanin signal, Cryptophyte like >2 µm with Chla and phycoerythrin signal, nanophytoplankton size 2-20 µm and microphytoplankton: size >20µm.

Zooplankton was collected with a plankton net (25 cm diameter, 50 µm mesh size) carefully dragged from the bottom of the mesocosm bags to the surface. Samples were immediately preserved with 30% EtOH and stored in 4°C. Zooplankton was identified to genus level using an inverted microscope at 10 and 40 x magnification.

### 2.7 Flowthrough measurement system, AquaBox

The flow through system AquaBox with a central unit control of automated sampling that introduce sequentially water from each mesocosm bag to a flow-through system, guiding the sample through a selected set of sensors, probes and analyzers. The system is highly flexible and different sensors could be added, but the setup we used is presented in Table 4. A more detailed description of the system is found in [14]. The raw data include measurements from the water in the tubes when changing from one mesocosm bag to another, and this data must be removed before data from individual mesocosms remain. We had some technical difficulties with the sensors monitoring dissolved gasses and here we include the optical measurements, which includes photosynthetic pigment fluorescence (Chla, phycoerythrin and phycocyanin), colored dissolved organic matter and photochemical efficiency, in addition to temperature and salinity. A mobile phone modem transferred the data real time to a database, enabling us to track the data stream from the laboratory.

## Ethics Statements

There is no ethical issue for this study as it only involves microscopic organisms and does not contain personal or otherwise sensitive data.

## CRediT authorship contribution statement

**Kristian Spilling:** Writing – original draft, Visualization, Formal analysis, Investigation. **Eero Asmala:** Formal analysis, Writing – review & editing. **Noora Haavisto:** Investigation, Writing – review & editing. **Lumi Haraguchi:** Investigation, Writing – review & editing. **Kaisa Kraft:** Investigation, Writing – review & editing. **Anne-Mari Lehto:** Investigation, Writing – review & editing. **Aleksandra Lewandowska:** Investigation, Writing – review & editing. **Joanna Norkko:** Supervision, Resources, Writing – review & editing. **Jonna Piiparinen:** Investigation, Writing – review & editing. **Jukka Seppälä:** Supervision, Investigation, Writing – review & editing. **Mari Vanharanta:** Investigation, Writing – review & editing. **Anu Vehmaa:** Investigation, Writing – review & editing. **Pasi Ylöstalo:** Investigation, Writing – review & editing. **Timo Tamminen:** Conceptualization, Investigation, Funding acquisition, Supervision.

## Declaration of Competing Interest

The authors declare that they have no known competing financial interests or personal relationships that could have appeared to influence the work reported in this paper.

## Data Availability

Brownification experiment in the Baltic Sea (Original data) (Mendeley Data).

## References

[1] Grasshoff K, Ehrhardt M, Kremling K. (1999).

[2] Koistinen J., Sjöblom M., Spilling K., Spilling K. (2019). Methods in Molecular Biology.

[3] Koistinen J., Sjöblom M., Spilling K., Spilling K. (2018). Methods in Molecular Biology.

[4] Koistinen J., Sjöblom M., Spilling K., Spilling K. (2018). Methods in Molecular Biology.

[5] Koistinen J., Sjöblom M., Spilling K., Spilling K. (2017). Methods in Molecular Biology.

[6] Koistinen J., Sjöblom M., Spilling K, Spilling K. (2017). Methods in Molecular Biology.

[7] Solórzano L., Sharp J.H. (1980). Determination of total dissolved phosphorus and particulate phosphorus in natural waters. Limnol. Oceanogr..

[8] Schindler D.W., Schmidt R.V., Reid R.A. (1972). Acidification and bubbling as an altemative to filtration in determining phytoplankton production by the ^14^C method. J. Fish. Res. Board. Can..

[9] Platt T., Gallegos C., Harrison W. (1980). Photoinhibition of photosynthesis in natural assemblages of marine phytoplankton. J. Mar. Res..

[10] Fuhrman J.A., Azam F. (1982). Thymidine incorporation as a measure of heterotrophic bacterioplankton production in marine surface waters: evaluation and field results. Mar. Biol..

[11] Smith D.C., Azam F. (1992). A simple, economical method for measuring bacterial protein synthesis rates in seawater using 3H-leucine. Mar. Microb. Food Webs.

[12] Helsinki Commission (HELCOM) (2008). Programme for monitoring of eutrophication and its effects. Annex C-11 guidelines concerning bacterioplankton growth determination. In Manual for marine monitoring in the COMBINE programme of HELCOM. Annex.

[13] Norland S., Kemp P.F., Sherr B.F., Sherr E.B., Cole J.J. (1993). Handbook of methods in aquatic microbial ecology.

[14] R. Ptacnik, T. Tamminen, P. Ylöstalo, S. Kielosto, J. Ruohola, J. Seppälä, A.M. Lehto, N. Aarnio, N. Haavisto, P. Kuuppo. Deliverable 8.3: Final Report AquaBox, AQUACOSM report series (2020).

[15] Spilling K., Asmala E., Haavisto N., Haraguchi L., Kraft K., Lehto A.M., Lewandowska A.M., Norkko J., Piiparinen J., Seppälä J., Vanharanta M., Vehmaa A., Ylöstalo P., Tamminen T. (2022). Brownification affects phytoplankton community composition but not primary productivity in eutrophic coastalwaters: Amesocosm experiment in the Baltic Sea. Sci. Tot. Env..

[16] Ma K., Powers L.C., Seppälä J., Norkko J., Brandes J.A. (2022). Effects of added humic substances and nutrients on photochemical degradation of dissolved organic matter in a mesocosm amendment experiment in the Gulf of Finland, Baltic Sea. Photochem. Photobiol..

